# Supplementary material for: Identification of photoperiod-induced specific miRNAs in the adrenal glands of Sunite sheep (Ovis aries)
Source: Front Vet Sci. 2022 Jul 22;9:888207. doi: 10.3389/fvets.2022.888207 (PMC9354845; doi:10.3389/fvets.2022.888207)
Supplement: Supplementary file 1 [file Data_Sheet_1.ZIP › Supplementary file/miRDeep2 file.zip!/miRDeep2 file/Novel_Structure/LP42A3/Novel_658.pdf]

Provisional ID	:	NC_019484.2_25330
Score total	:	1.9
Score for star read(s)	:	-1.3
Score for read counts	:	0
Score for mfe	:	1.6
Score for randfold	:	1.6
Score for cons. seed	:	
Total read count	:	1066
Mature read count	:	1066
Loop read count	:	0
Star read count	:	0

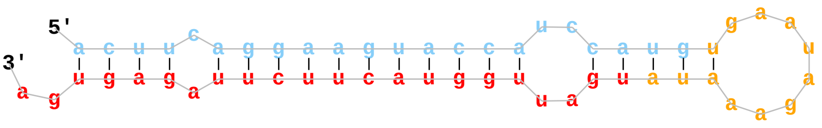

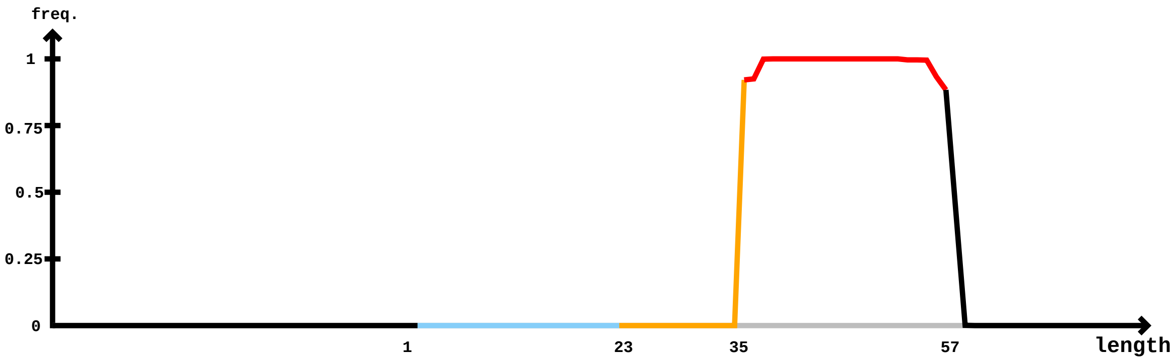

Star Mature

```
5'- ggucaccacauuucacuaugccgcgugcaguauugcacuucaggaaguaccauccaugugaauagaaauaugauugguacuucuuagagugaaguaaugcauagcaaauaca   -3'    exp
    (((...............)))(((((((.((((.(((((.(((((((((((..(((((........)))))..))))))))))).))))).)))).))))).)).......         reads      mm         sample
    ......................................................................ugauugguacuucuuag.........................         4          0          seq
    ......................................................................ugauugguacuucuuagag.......................         1          0          seq
    ......................................................................ugauugguacuucuuagagu......................         66         0          seq
    ......................................................................ugauugguacuucuuagagug.....................         51         0          seq
    ......................................................................ugauugguacuucuuagaguga....................         748        0          seq
    ......................................................................ugauugguacuucuuagagugaa...................         111        0          seq
    ......................................................................ugauugguacuucuuagagugaag..................         1          0          seq
    .......................................................................gauugguacuucuuagagug.....................         1          0          seq
    .......................................................................gauugguacuucuuagaguga....................         3          0          seq
    ........................................................................auugguacuucuuagagug.....................         1          0          seq
    ........................................................................auugguacuucuuagaguga....................         51         0          seq
    ........................................................................auugguacuucuuagagugaa...................         27         0          seq
    .........................................................................uugguacuucuuagaguga....................         1          0          seq
```
